# Complete genome sequence of the salmonella enterica serovar enteritidis bacteriophages fSE1C and fSE4C isolated from food matrices

**DOI:** 10.1186/s40793-016-0218-y

**Published:** 2017-01-05

**Authors:** Javier Santander, Jose I. Vasquez, Cristopher Segovia, Leonardo Santos, Gabriel Turra, Karen Huber, James Robeson

**Affiliations:** 1 Department of Ocean Sciences, Memorial University of Newfoundland, St. John’s, Canada; 2 Universidad Mayor, Faculty of Sciences, Huechuraba, Chile; 3 Pontificia Universidad Católica de Valparaiso, Institute of Biology, Valparaiso, Chile; 4 Memorial University of Newfoundland, Faculty of Sciences, Ocean Science Centre, Marine Microbial Pathogenesis and Vaccinology Laboratory AX3005, 0 Marine Lab Rd, Logy Bay, NL A1K 3E6 Canada

**Keywords:** *Salmonella enterica* serovar Enteritidis, Bacteriophages *f*SE1C and *f*SE4C, Shelf stability, Phage prophylaxis, Food security

## Abstract

*Salmonella enterica* serovar Enteritidis is one of the most common causes of Salmonellosis worldwide. Utilization of bacteriophages as prophylactic agents is a practical solution to prevent Salmonellosis in ready-to-eat products. Shelf stability is one of the desirable properties for prophylactic bacteriophages. Here, we describe the phenotype, genome, and phylogeny of *f*SE1C and *f*SE4S *Salmonella* bacteriophages. *f*SE1C and *f*SE4S were previously isolated from pickle sauce and ground beef respectively and selected for their significant shelf stability. *f*SE1C and *f*SE4S showed a broad *S. enterica* serovar range, infecting several *Salmonella* serovars. The viral particles showed an icosahedral head structure and flexible tail, a typical morphology of the *Siphoviridae* family. *f*SE1C and *f*SE4C genomes consists of dsDNA of 41,720 bp and 41,768 bp with 49.73% and 49.78% G + C, respectively. Comparative genomic analysis reveals a mosaic relationship between *S. enterica* serovar Enteritidis phages isolated from Valparaiso, Chile.

## Introduction

The current methodologies to inactivate bacterial pathogens in ready-to-eat products are not infallible. Foodborne diseases caused by non-typhoid *Salmonella* still have an enormous impact on public health [1, 2]. *Salmonella enterica* serotype Enteritidis is one of the most common causes of non-typhoid Salmonellosis with contaminated food [3–5]. The increasing cases of Salmonellosis together with the emergence of antibiotic resistant strains have led to efforts searching for new methods to control *Salmonella* colonization in ready-to-eat products. Traditional methods to reduce bacterial contamination (U.V., steam, and dry heat) face the problems of food organoleptic properties deterioration and lack of prophylactic protection once the product is contaminated. Also, some of these approaches used in the food industry to reduce contamination by food borne pathogens cannot be directly applied to fresh fruits, vegetables, and raw meat [6]. Despite technical advances to avoid transmission of bacterial pathogens throughout the food chain, novel strategies are still required to fulfill consumer demands to minimize chemical preservatives in fresh food products. Bacteriophage-based biocontrol has a great potential to enhance microbiological safety based on their long history of safe use, relatively easy handling, high and specific antimicrobial activity and public acceptance [7].

Shelf stability is one of the desirable characteristics that a bacteriophage must have for its effective utilization in fresh food [6]. Previously, we isolated the bacteriophages *f*SE1C and *f*SE4S from pickle sauce and ground beef respectively [8]. These bacteriophages have a significant stability in shelf conditions and in food matrices with respect to other *Salmonella* bacteriophages [8], making *f*SE1C and *f*SE4S excellent candidates to be used in ready-to-eat products. Here, we report the phenotypic characteristics, genome sequence, and phylogeny of *f*SE1C and *f*SE4S bacteriophages isolated from food matrices in Valparaiso, Chile.

## Organism information

### Classification and features

**Fig. 1:**
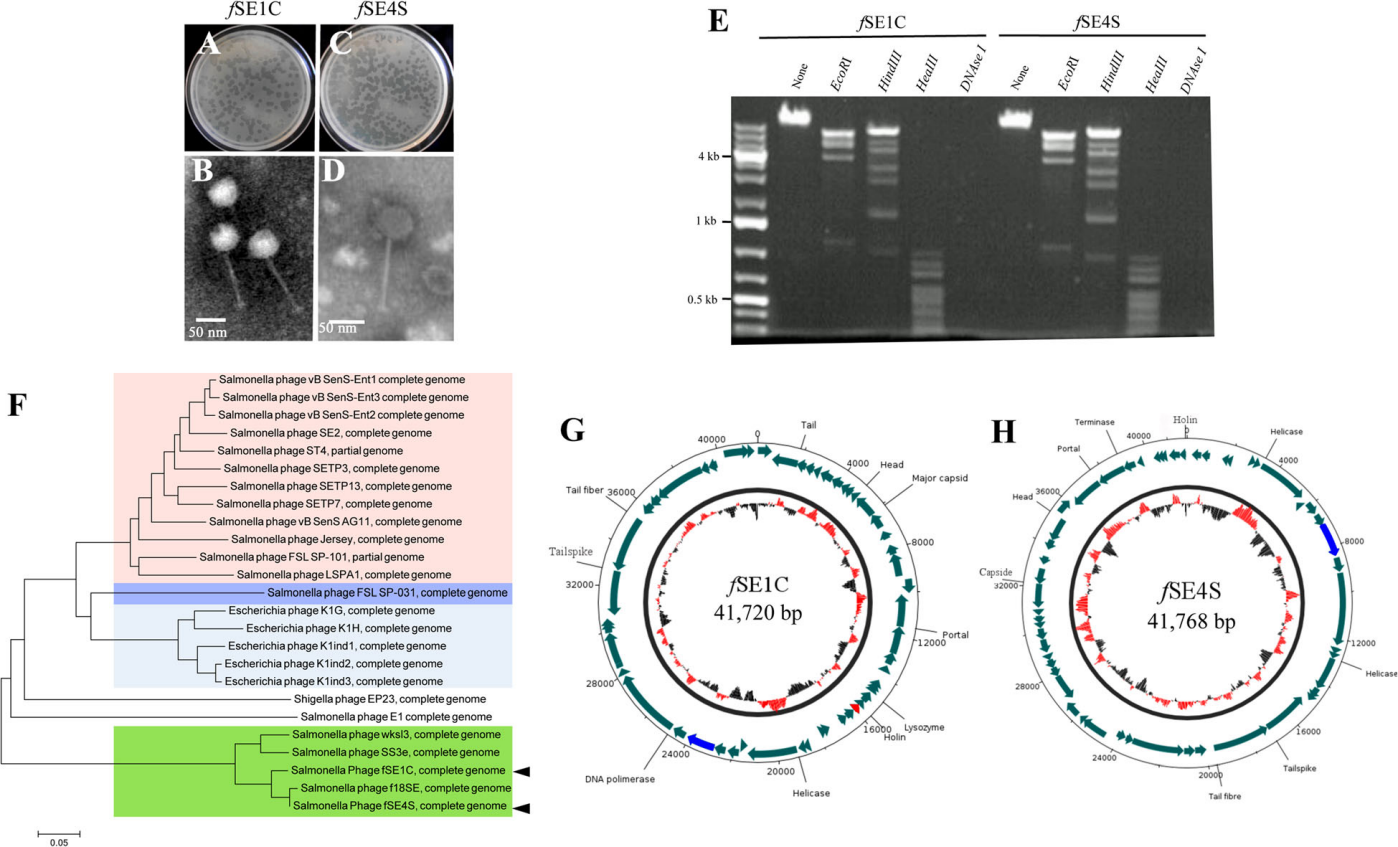
Bacteriophage characterization. **a.** Lysis halo of *f*SE1C on *S.* Enteritidis lawn; **b.** TEM of fSE1C; **c.** Lysis halo of *f*SE4S on *S.* Enteritidis lawn; **d.** TEM of fSE4S; **e.** Restriction pattern of bacteriophage genomic DNA; **f.** Evolutionary relationships of *f*SE1C and *f*SE4S bacteriophages; light red: *Jerseyvirus*; violet: *Sp3unalikevirus*; blue: *K1glikevirus*; green: current isolated phages members of the *Jerseyvirus* genus; The evolutionary history was inferred using the Neighbor-Joining method [23]. The optimal tree with the sum of branch length = 2.55835582 is shown. The tree is drawn to scale, with branch lengths in the same units as those of the evolutionary distances used to infer the phylogenetic tree. The evolutionary distances were computed using the *p*-distance method [25] and are in the units of the number of base differences per site. The analysis involved 25 nucleotide sequences. All ambiguous positions were removed for each sequence pair. There were a total of 104441 positions in the final dataset. Evolutionary analyses were conducted in MEGA6 [26]. **g.** *f*SE1C bacteriophage genome map; the unique gene to *f*SE1C is indicated in red and the putative *cas4* gene in blue; **h.** *f*SE4S bacteriophage genome map; the putative *cas4* gene is indicated in blue. The internal circle show the G + C % in red and the A + T % in black. DNAPlotter was utilized for genome map visualization [33]

The bacteriophages *f*SE1C and *f*SE4S were isolated from pickle sauce and ground beef respectively, from samples obtained at the Central Market of Valparaiso, Chile, during 2013. Routine enrichment techniques [9] and the host, *S. enterica* serovar Enteritidis PT4 [8] were utilized for the isolation process. The two phages isolated formed clear plaques on the host bacterial lawn after 18 h of incubation at 37 °C. The diameters of plaques were 1 mm for both phages (Fig. 1a and b). *f*SE1C and *f*SE4S showed a productive lytic infection in different *S. enterica* serovars including *S. enterica* serovar Enteritidis (control), *S. enterica* serovar Infantis, *S. enterica* serovar Heidelberg, *S. enterica* serovar Typhi, *S. enterica* serovar Typhimurium, *S. enterica* serovar Paratyphi B and *S. enterica* serovar Pullorum. The bacteriophages have a different host range. *f*SE4S can have a productive lytic infection in *S. enterica* serovar Derby and *S. enterica* serovar Hadar in contrast to *f*SE1C [10]. The transmission electron microscopy showed that these bacteriophages have a typical morphology of the *Siphoviridae* family consisting of an icosahedral head (~50 nm), flexible long non-contractile tail (~150 nm) and base (Fig. 1b and d). The extracted nucleic acids from phage particles were treated with *EcoR*I, *Hind*III and *HaeI*II restriction enzymes. The genomic material of both phages was digested by these enzymes, revealing that their genomic material is dsDNA (Fig. 1e). The restriction enzyme patterns were similar for both phages (Fig. 1e). Taken together, these results indicated these phages belong to the *Siphoviridae* family [11]. Phylogenetic analysis, using the complete bacteriophage genomes, showed that these phages are close related to *f*18SE [12], SSe and wksl3 *Salmonella* phages (Fig. 1f). The bacteriophage SSe, wksl3 and *f*18SE are members of the proposed subfamily *Jersyvirinae* [12], genera *Jersylikekvirus* [13]. However our phylogenetic analysis, which includes the most recently sequenced *Salmonella* *Siphoviridae* bacteriophages, revealed that *f*SE1C, *f*SE4S, *f*18SE, SSe and wksl3 are distant members from the *Jersylikekvirus* genera (Fig. 1f).

**Fig. 2:**
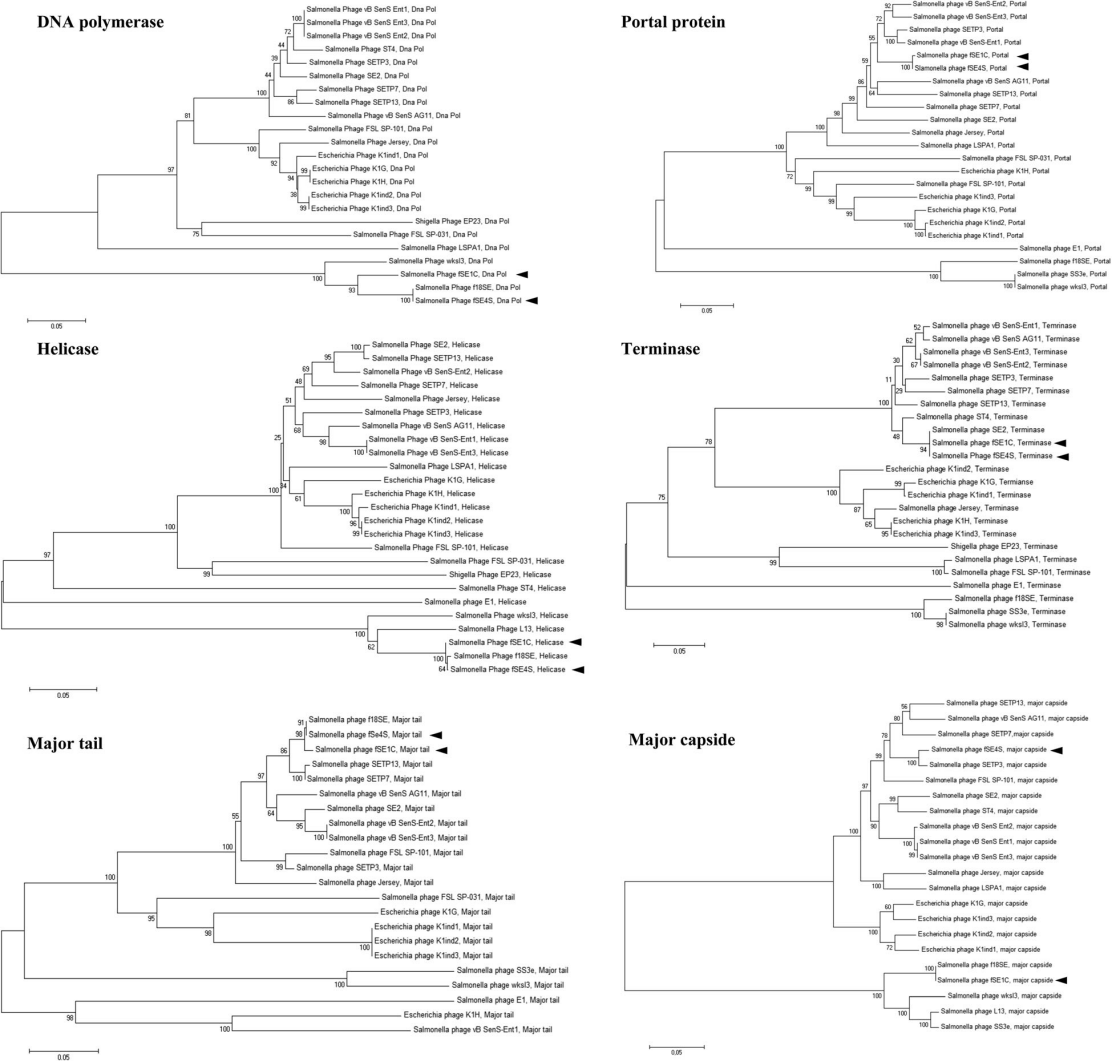
Phylogenetic analysis of conserved genes of *Siphoviridae* bacteriophages. Phylogenetic tree of conserved gene on bacteriophages of *Siphoviridae* family, and *f*SE1C and *f*SE4S. The evolutionary history was inferred using the Neighbor-Joining method [23]. DNA Polymerase, helicase, major tail, portal protein, terminase, and major capside gene sequences were selected. The percentage of replicate trees in which the associated taxa clustered together in the bootstrap test (1000 replicates) are shown next to the branches [24]. The evolutionary distances were computed using the p-distance method [25] and are in the units of the number of base differences per site. Evolutionary analyses were conducted in MEGA6 [26]

Genes encoding DNA polymerase, helicase, the major tail protein, portal protein, the terminase large subunit and the major capsidase, were predicted from the genomes of both phages and used for phylogenetic analysis (Fig. 1g and h). DNA polymerase, helicase and the major tail protein are closely related to the bacteriophage *f*18SE [12] (Fig. 2). On the other hand, the portal protein and the terminase large subunit are closely related between both phages, but not related to the *f*18SE bacteriophage (Fig. 2). The major capsid subunit of the phage *f*SE1C is closely related to *f*18SE, in contrast to *f*SE4S, which is closely related to the SETP3 phage (Fig. 2). Mosaicism is known to be prevalent in the family *Siphoviridae*, which is reflected in our results. However, the DNA polymerase, and helicase proteins presented similar phylogenic relationships, analogous to the complete bacteriophage genome phylogenic relationships (Fig. 1f). Information on the isolation, classification, and general features of the phages *f*SE1C and *f*SE4S are presented in Table 1.

**Table 1:** Classification and general features of *Salmonella enterica* bacteriophages *f*SE1C and *f*SE4S

MIGS ID	Property	Term *f*SE1C and *f*SE4S	Evidence code^a^
	Classification	Domain Akamara	TAS [34]
		Kingdom Viruses	TAS [34]
		Class dsDNA viruses, no RNA stage	IDA
		Order Caudovirales	TAS [34]
		Family Siphoviridae	TAS [34]
		Genus *Jerseyvirus*	TAS [34]
		Species *Salmonella* phage	TAS [34]
		Strains: *f*SE1C, *f*SE4S	TAS [34]
	Gram stain	Not applicable	TAS [34]
	Particle shape	Icosahedral head with a flexible long non-contractile tail	IDA
	Motility	none	TAS [34]
	Sporulation	none	NAS
	Temperature range	−80 °C – 45 °C	TAS [31]
	Optimum temperature	37 °C	TAS [34]
	pH range; Optimum	3.5–6.5; 7.0	TAS [34]
	Carbon source	Not applicable	TAS [34]
MIGS-6	Habitat	Contaminated food or waste water	IDA
MIGS-15	Biotic relationship	intracellular parasite of *Salmonella enterica*	IDA
MIGS-14	Pathogenicity	virulent phage of *Salmonella enterica*	IDA
MIGS-4	Geographic location	Mercado Cardonal, Valparaiso, Chile	IDA
MIGS-5	Sample collection	2013	IDA
MIGS-4.1	Latitude	33°2′S	IDA
MIGS-4.2	Longitude	71°40′W	IDA
MIGS-4.4	Altitude	0 m	IDA

^a^Evidence codes – *IDA* Inferred from Direct Assay, *TAS* Traceable Author Statement, *NAS* Non-traceable Author Statement. These evidence codes are from Gene Ontology project [35]

## Genome sequencing information

### Genome project history

Genome sequencing of the bacteriophages *f*SE1C and *f*SE4S was performed as a part of a research project that aimed to sequence effective bacteriophages fore use in anti-*Salmonella* prophylactic cocktails for ready-to-eat products. Previously, we reported the genome sequence of the *Salmonella* bacteriophage *f*18SE isolated from the poultry industry in Valparaiso, Chile, during 2001, which has been tested successfully in vivo and in processed foods [14–16] as part of this project.

Genome sequencing of *f*SE1C and *f*SE4S was performed using the NGS Illumina MiSeq at Universidad Mayor, Center for Genomics and Bioinformatics (Huechuraba, Chile). The sequences were assembled using CLC Genomics Workbench 8.5.1 (Qiagen), resulting in single contigs. The assembled sequences were annotated by the PHASTER server [17, 18] and the NCBI-PGAAP. The complete genome sequences and annotation information of both bacteriophages were submitted to GenBank under the accession numbers KT962832 (*f*SE1C) and KT881477 (*f*SE4S) (Table 2).

**Table 2:** Project information of *Salmonella enterica* bacteriophages *f*SE1C and *f*SE4S

MIGS ID	Property	Term *f*SE1C	Term *f*SE4S
MIGS 31	Finishing quality	Finished	Finished
MIGS-28	Libraries used	1	1
MIGS 29	Sequencing platforms	One paired-end Illumina library, MiSeq	One paired-end Illumina library, MiSeq
MIGS 31.2	Fold coverage	2874X	7590X
MIGS 30	Assemblers	CLC Genome Workbench 8.5.1	CLC Genome Workbench 8.5.1
MIGS 32	Gene calling method	RAST version 2.0, GeneMark.hmm, and GLIMMER	RAST version 2.0, GeneMark.hmm, and GLIMMER
	Locus Tag	*f*SE1C	*f*SE4S
	Genbank ID	KT962832	KT881477
	GenBank Date of Release	18-NOV-2015	31-JUL-2016
	GOLD ID	952094059	952094006
	BIOPROJECT	PRJNA291403	PRJNA291403
MIGS 13	Source Material Identifier	NA^a^	NA^a^
	Project relevance	Phage prophylaxis in ready-to-eat products	Phage prophylaxis in ready-to-eat products

^a^Viruses have not been deposited yet

### Growth conditions and genomic DNA preparation

The bacteriophages *f*SE1C and *f*SE4S were isolated from pickle sauce and ground beef respectively using *S. enterica* serovar Enteritidis PT4 as host [8]. Isolation and propagation methods were those used routinely [9, 19]. Briefly, the bacteriophages were enriched using a *S. enterica* serovar Enteritidis PT4 Rif^r^, Nal^r^ derivative. Lysis plaques were obtained by under streaking using the same bacterial host. Individual plaques were purified twice to establish the final bacteriophage culture typified by the formation of clear, haloed round plaques of about 1 mm in diameter. Both phages showed similar plaque morphology. The two phages formed clear plaques on *S. enterica* serovar Enteritidis lawn after 18 h incubation at 37 °C. Genomic DNA from concentrated lysates were purified according to the method described by Kaiser et al. [20].

### Genome sequencing and assembly

The purified bacteriophage DNA was used to prepare the libraries (one library for each phage) with the Nextera kit (Illumina, San Diego, CA). High-throughput sequencing of the libraries was performed using a MiSeq (Illumina) with a 2x300bp paired-end run, with the reagent kit version 3 (600 cycles) at the Center for Genomics and Bioinformatics, Universidad Mayor, Chile. In total, about 127 and 317 million pairs of reads were obtained for *f*SE1C and *f*SE4S, respectively. Raw reads were assembled by using CLC Genomics Workbench 8.5.1. Coverage was calculated from the sequencing statistics, and final contig sizes were 2874× and 7590× for *f*SE1C and *f*SE4S, respectively (Table 2).

### Genome annotation

Contigs were annotated using a combination of automatic annotations by the PHASTER server [17, 18], and the NCBI PGAAP. Functional annotation of protein coding genes was improved by RPS-BLAST searches against the CDD [21]. Signal sequence peptides and transmembrane helices were predicted by the Phobius software [22]. BLASTp searches against the NCBI nr database were also performed. The CRISPRs were predicted base on structure using the web base software Structure RNA finder.

The evolutionary history was inferred using the Neighbor-Joining method [23]. The trees were drawn to scale. The percentage of replicate trees for the conserved proteins in the bootstrap test (1000 replicates) are shown next to the branches [24] (Fig. 2). The evolutionary distances were computed using the *p*-distance method [25] and are in the units of the number of base differences per site. The ambiguous positions were removed for each sequence pair. Evolutionary analyses were conducted in MEGA6 [26].

## Genome properties

The complete genomes of both phages were assembled into single circular contigs. Bacteriophage *f*SE1C contains 41,720 bp and has a G + C content of 49.73%. The bacteriophage *f*SE4S contains 41,768 bp and has a G + C content of 49.78%. The genome of *f*SE1C contains 53 predicted genes and *f*SE4S contains 52 predicted genes, with a total gene length between 186–3099 bp. We found in *f*SE1C genome 17 genes with rightward orientation, while 36 were leftward oriented, and in *f*SE4S genome 35 genes with rightward orientation and 17 were leftward (Fig. 1g and h) (Table 3). Both phage genomes contain genes for replication, structure, and lysis. Open reading frames (ORFs) were found for putative homing endonuclease, helicase, and DNA polymerase. The ORFs for terminase (large and small subunit), head morphogenesis protein, major capside protein, putative tail protein, and tail fiber protein and a portal protein were found. Also, a lysozyme, holing-like classes I and putative endolysins were also found. Lysogeny related genes, like C2 of P22 [27], CI and Cro of λ [28], and others are absent from both phage genomes.

**Table 3:** Genome statistics

Attribute	Value *f*SE1C	% of Total *f*SE1C	Value *f*SE4S	% of Total *f*SE4S
Genome size (bp)	41,720	100.00	41,768	100.00
DNA coding (bp)	36,813	88.24	37,032	88.66
DNA G + C (bp)	20,747	49.73	20,926	49.78
DNA scaffolds	1	100.00	1	100.00
Total genes	53	88.24	52	88.66
Protein coding genes	53	88.24	52	88.66
RNA genes	0	0.00	0	0.00
Pseudo genes	0	0.00	0	0.00
Genes in internal clusters	0	0.00	0	0.00
Genes with function prediction	22	36.62	18	30.69
Genes assigned to COGs	10	19.98	26	20.46
Genes with Pfam domains	31	36.36	33	52.26
Genes with signal peptides	0	0.00	0	0.00
Genes with transmembrane helices	0	0.00	0	0.00
CRISPR direct repeats	2	0,24	2	0,24

The total is based on the size of the genome in base pairs

The phage genomes closely related to *f*SE1C and *f*SE4S were *Salmonella* phages *f*18SE (GenBank accession no. KR270151), SSe3 (GenBank accession no. AY730274), and wsk13 (GenBank accession no. JX202565). Comparative analysis between both phages showed that their genomes are 43.09% similar and all 52 genes of *f*SE4S have orthologous in the *f*SE1C genome. These orthologous proteins have a similarity between 73.58 and 100%. The only gene different in the *f*SE1C genome encodes for a hypothetical protein (GI:952094085) of 108 aa with no ortholog in *f*SE4S, but present in *f*18SE and other lytic *Salmonella* bacteriophages.

Non-coding RNA prediction was similar in both bacteriophages, presenting the CRISPR-DR41 and CRISPR-DR23 single direct repeat. This prediction was coincident with the COGs analyses (Table 4), which detected the Cas4 protein family (cl00641) in both bacteriophages. Functional CRISPRs have been described in *V. cholerae* bacteriophages [29], however, the CRISPRs predicted for *f*SE1C and *f*SE4S seem not a completed CRISPR system.

**Table 4:** Number of genes associated with general COG functional categories

Code	*f*SE1C		*f*SE4S		Description
	Value	%age	Value	%age	
J	1	1.89	1	1.92	Translation, ribosomal structure and biogenesis
A	0	0	0	0	RNA processing and modification
K	2	3.78	11	21.12	Transcription
L	5	9.45	19	36.48	Replication, recombination and repair
B	0	0	0	0	Chromatin structure and dynamics
D	0	0	0	0	Cell cycle control, Cell division, chromosome partitioning
V	1	1.89	1	1.92	Defense mechanisms
T	0	0	0	0	Signal transduction mechanisms
M	0	0	0	0	Cell wall/membrane biogenesis
N	0	0	0	0	Cell motility
U	0	0	0	0	Intracellular trafficking and secretion
O	0	0	1	1.92	Posttranslational modification, protein turnover, chaperones
C	0	0	0	0	Energy production and conversion
G	0	0	0	0	Carbohydrate transport and metabolism
E	0	0	0	0	Amino acid transport and metabolism
F	0	0	0	0	Nucleotide transport and metabolism
H	0	0	0	0	Coenzyme transport and metabolism
I	0	0	0	0	Lipid transport and metabolism
P	0	0	1	1.92	Inorganic ion transport and metabolism
Q	0	0	5	9.6	Secondary metabolites biosynthesis, transport and catabolism
R	0	0	2	3.84	General function prediction only
S	3	4.67	10	19.2	Function unknown
-	43	81.27	23	44.16	Not in COGs

The total is based on the total number of protein coding genes in the genome

## Conclusions

The ORFs involved in structure, replication, host specificity (i.e., tail fibers and tailspikes) and DNA metabolism were found to be conserved in these two phages compared to other *Salmonella enterica* bacteriophages. However, the major capsid protein showed some diversity (Fig. 2) that might be related to the high shelf stability presented by *f*SE1C and *f*SE4S phages [8].

The *Jersyvirine* subfamily consists of three genera, “*Jerseyvirus*”, “*Sp3unavirus*” and “*K1gvirus*” [13]. The *Jersyvirine* subfamily include a distinct morphotype, genomes of 40–44 kb (49.6-51.4 mol % G + C), a syntenic genome organization, high degree of nucleotide sequence identity, and strictly lytic cycle [30]. As mentioned previously, the *Siphoviriade* family presents considerable mosaicism [31, 32] and although we distinguished a possible new genus for the subfamily *Jersyvirinae* (Fig. 1f), we considered that a high number of sequenced *Jersyvirinae* phages are required to propose a new genus.

## Abbreviations

CDD: Conserved domain database
CRISPRs: Clustered regularly interspaced short palindromic repeats
DR: Direct repeats
MEGA: Molecular evolutionary genetics analysis
NGS: Next generation sequencer
PGAAP: Prokaryotic genomes automatic annotation pipeline
PHASTER: PHAge search tool enhanced release
TEM: Transmission electron microscopy
